# Personalized Three-Dimensional Printed Models in Congenital Heart Disease

**DOI:** 10.3390/jcm8040522

**Published:** 2019-04-16

**Authors:** Zhonghua Sun, Ivan Lau, Yin How Wong, Chai Hong Yeong

**Affiliations:** 1 Discipline of Medical Radiation Sciences, School of Molecular and Life Sciences, Curtin University, Perth 6102, Australia; ivan.w.lau@curtin.edu.au; 2 School of Medicine, Faculty of Health and Medical Sciences, Taylor’s University, Subang Java 47500, Malaysia; YinHow.Wong@taylors.edu.my

**Keywords:** three-dimensional printing, congenital heart disease, medical education, heart models, pre-operative planning, simulation

## Abstract

Patient-specific three-dimensional (3D) printed models have been increasingly used in cardiology and cardiac surgery, in particular, showing great value in the domain of congenital heart disease (CHD). CHD is characterized by complex cardiac anomalies with disease variations between individuals; thus, it is difficult to obtain comprehensive spatial conceptualization of the cardiac structures based on the current imaging visualizations. 3D printed models derived from patient’s cardiac imaging data overcome this limitation by creating personalized 3D heart models, which not only improve spatial visualization, but also assist preoperative planning and simulation of cardiac procedures, serve as a useful tool in medical education and training, and improve doctor–patient communication. This review article provides an overall view of the clinical applications and usefulness of 3D printed models in CHD. Current limitations and future research directions of 3D printed heart models are highlighted.

## 1. Introduction

Computed tomography (CT), magnetic resonance imaging (MRI), and echocardiography represent commonly used imaging modalities in the diagnostic assessment of congenital heart disease (CHD). These imaging techniques allow for generation of two-dimensional (2D) and three-dimensional (3D) visualizations, which play an important role in understanding the complexity of CHD and assisting pre-procedural planning of cardiac procedures. Despite useful information provided by these imaging modalities, the images are still limited to be viewed on 2D screens which is very different from the physical models that offer realistic visualization of 3D spatial relationship between normal and anomalous anatomy. 3D printing overcomes this limitation by creating patient-specific or personalized medical models [1,2,3]. The tactile experience offered by 3D printed models is another advantage over traditional image visualizations as the physical models enable comprehensive evaluation of anatomical and pathological structures which cannot be obtained by other methods [4].

3D printing has been increasingly utilized in the medical field with studies confirming its clinical value and usefulness in many areas, ranging from medical education to pre-surgical planning and simulation of complex surgeries, and to patient–doctor communication [5,6,7,8,9,10]. In particular, personalized 3D printed models have been shown to offer valuable information for treating patients with CHD due to complexity and anatomic variation associated with this disease. Most of the current reports on 3D printing in CHD are dominated by isolated case reports or case series, with only a few single or multi-center studies and randomized controlled trials (RCT) available in the literature. This review aims to provide an in-depth overview of the current applications of 3D printed models in CHD, with limitations and future directions briefly highlighted.

## 2. Image Post-Processing and Segmentation Process for Three-Dimensional (3D) Printing in Congenital Heart Disease (CHD)

**Figure 1:**
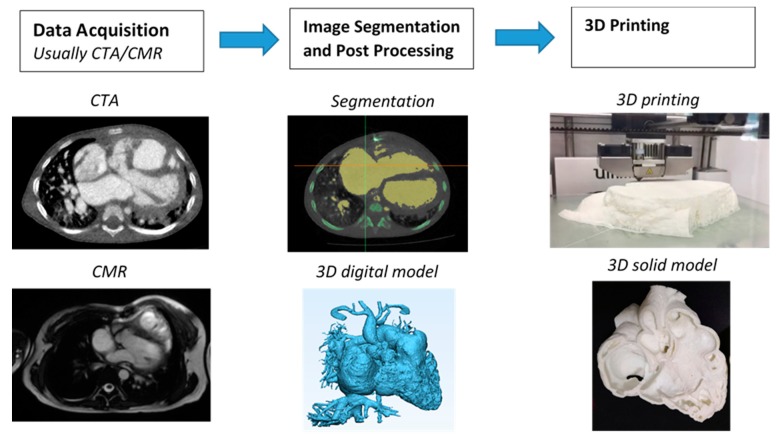
Steps involved in fabrication of 3D printed heart models. CTA—computed tomography angiography; CMR—cardiac magnetic resonance; 3D—three-dimensional.

The first step to generate a 3D printed heart model is to undergo a series of image post-processing and segmentation of volumetric data, which are commonly acquired with cardiac CT or MRI imaging. While high-resolution original datasets are important for accurately isolating the desired anatomy of interest and pathology from surrounding structures, segmentation of cardiac structures remains challenging due to complexity of cardiac features, especially in the CHD cases. Different software is used for segmentation, with Mimics (Materialise HQ, Leuven, Belgium) being the most commonly used commercial software and 3D Slicer (Brigham and Women’s Hospital, Boston, Mass) as the most common open-source tool. Several review articles have provided excellent description of details about the steps required from data acquisition to image post-processing and segmentation [11,12,13,14]. Figure 1 shows the steps to create 3D printed models from data acquisition to image post-processing and segmentation.

## 3. Accuracy of 3D Printed Heart Models

The most important part of creating 3D printed models is to ensure that 3D models accurately delineate anatomical structures and pathologies since the model accuracy is essential for treatment planning [15]. Current research evidence indicates that 3D printed heart models are generally accurate [16], and this has been validated by other studies, either based on case reports/series or single- or multi-center studies [17,18,19,20,21,22,23]. In most of the studies, model accuracy is determined by the degree of agreement between the measured dimensions of the 3D printed model and the dimensions of original source images, usually using cardiac CT, MRI, and sometimes using rotational angiography or echocardiography [16,17,18,22], or intraoperative findings [19]. Currently, there is no standardized method to measure the dimensions of the 3D printed heart models. Most of the studies carried out measurement using calipers on the physical 3D printed models [17,20,21]. Only a few studies conducted measurement on the standard tessellation language (STL) file [18] and conducted CT scan on the 3D printed model for measurement [16]. The authors claimed it is easier to replicate the exact plane for measurement comparison, hence improving the accuracy of the results [16,18].

**Figure 2:**
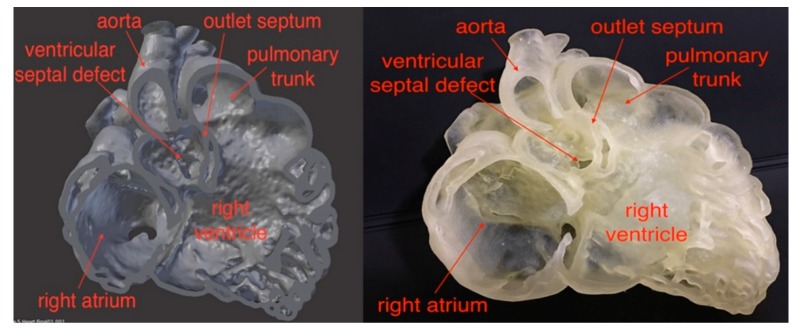
Comparison of virtual 3D reconstruction model (left) and 3D printed heart model (right). Reprinted with permission under the open access from Lau et al. [16].

**Figure 3:**
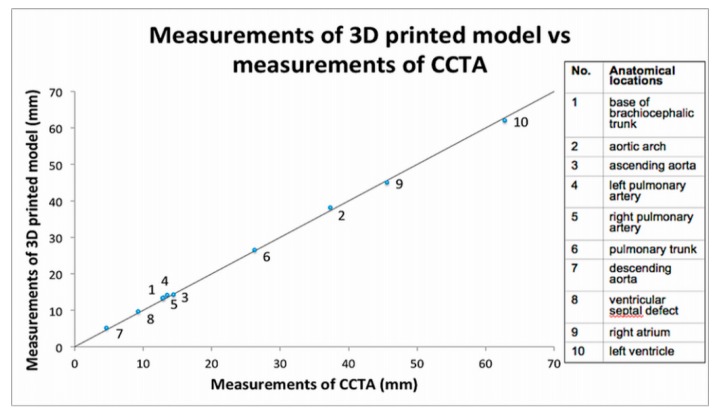
Scatterplot showing measurements of 3D printed model in comparison with those from cardiac computed tomography (CT) images at 10 anatomical locations. CCTA—cardiac computed tomography angiography. Reprinted with permission under the open access from Lau et al. [16].

Despite limited studies available in the literature regarding quantitative assessment of 3D printed heart models, the accuracy of 3D printed heart models is within 1 mm in terms of dimensional differences when compared to the original images. Lau et al. compared model accuracy between contrast-enhanced CT images of the 3D printed heart model (Figure 2 and Figure 3) and original cardiac CT images in 10 anatomical locations including ventricular septal defect (VSD) [16]. High accuracy was achieved between these measurements by only 0.23 mm difference in average. Ma et al. in their study comprising 35 patients of Tetralogy of Fallot (ToF), compared measurements of VSD sizes between 3D printed models and intraoperative findings with no significant differences found (mean value ± standard deviation: 14.98 ± 1.91 vs. 15.11 ± 2.06 mm, *p* > 0.05) [19]. This is further confirmed by a multi-center study showing the model accuracy. Valverde et al. recruited 40 patients diagnosed with complex CHD from 10 international centers in their prospective study [21]. 3D printed models were created from CT or MRI images, and they were found to be highly accurate with mean differences of 0.27 ± 0.73 mm between measurements performed on the 3D printed models and CT/MRI images.

Currently, there is a lack of measurement comparison between 3D printed heart models and STL file, hence, it is unknown whether there is any dimensional error introduced during 3D printing process. This needs to be addressed by future studies.

## 4. 3D Printed Models in Medical Education and Training

3D printed heart models have been shown to serve as a novel teaching tool in medical education and training and this is confirmed by RCT available in the literature [22,23,24,25]. Three of them reported the usefulness of 3D printed models of different types of CHD in medical education [22,23,24]. Table 1 shows details of these three studies and other single- and multi-center reports.

**Figure 4:**
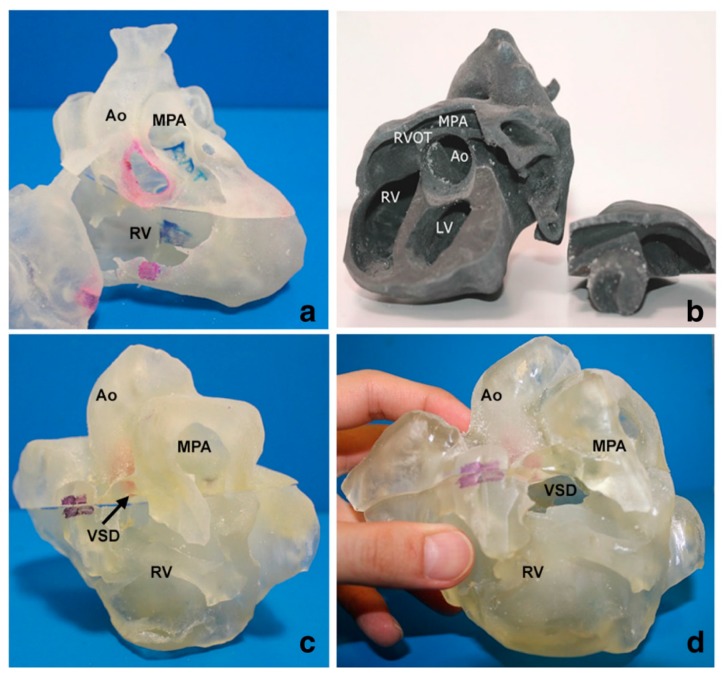
3D printed heart models showing normal anatomy and pathology. (**a**) Normal heart model created from cardiac CT and is partitioned into three pieces allowing visualization of interventricular septum. (**b**) Repaired Tetralogy of Fallot (ToF) from an adult patient. The model was created from cardiac magnetic resonance imaging (MRI) and separated into two pieces allowing for clear visualization of overriding aorta and pulmonary infundibular stenosis. (**c**) Unrepaired ToF heart model from an infant. The model was created from 3D echocardiographic images and partitioned into two pieces showing the ventricular septal defect (VSD). (**d**) Unrepaired ToF heart model from an infant with superior and inferior portions showing VSD and the aortic overriding in relation to the VSD. Reprinted with permission under the open access from Loke et al. [22]. Ao—Aorta; MPA—Main Pulmonary Artery; LV—Left Ventricle; RV—Right Ventricle; RVOT—Right Ventricular Outflow Tract; VSD—Ventricular Septal Defect; ToF—Tetralogy of Fallot.

**Figure 5:**
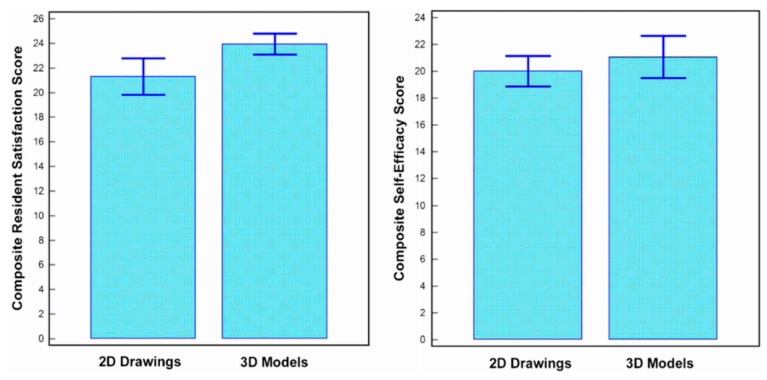
Impact of 3D printed heart models on medical education. Improvement was found in residents’ knowledge on congenital heart disease with use of 3D printed models when compared to 2D images. A statistically significant difference was noticed in satisfaction ratings in the group having 3D printed heart models when compared to the control group (*p* = 0.03). While residents in the 3D printed model group had higher self-efficacy scores, this did not reach significant difference compared to the control group using 2D images/drawings (*p* = 0.39). Reprinted with permission under the open access from Loke et al. [22].

Studies by Loke et al. and White et al. investigated how 3D printed models improved pediatric residents’ knowledge and understanding of CHD, while the study by Su et al. focused on how 3D printed models improved medical students’ knowledge in CHD. In these studies, 3D printed models of VSD and ToF representing simple and complex CHD were created and used for teaching in the test groups (Figure 4), while the control groups were only given the usual lectures with 2D images. Overall results showed significant improvements of residents and medical students’ learning and confidence in managing CHD, especially in dealing with complex CHD situations such as ToF as confirmed by White et al. [24]. Furthermore, 3D printed models significantly improved residents’ satisfaction and self-efficiency scores when compared to learning from 2D images [22] (Figure 5).

Results from cross-sectional studies are consistent with these findings from RCT [5,6,30,31,32]. To date, there is sufficient evidence to prove that 3D printed models of CHD play a valuable role in education and training of medical students, pediatric residents, and healthcare professionals in improving their understanding of complex cardiac pathology and increasing their confidence in managing CHD patients.

## 5. 3D Printed Models in Pre-Surgical Planning and Simulation

Due to complexity of the cardiac conditions with wide variations between individuals with CHD, 3D printed models demonstrate great advantages over traditional image visualizations in pre-surgical planning and simulation of cardiac surgeries. A recent systematic review has summarized findings from a number of case reports and series with regard to the use of 3D printed models in facilitating preoperative planning and surgical decision-making in CHD cases [33]. Table 1 shows some results from single- and multi-center studies which involved more than 20 cases or participants about the value of 3D printed heart models in this aspect [20,26,27,28,29]. These studies reported the usefulness of 3D printed heart models from different perspectives. Among all types of CHD, double outlet right ventricle (DORV) and ToF represent the most common types of CHD for fabrication of 3D printed models. This is reported in four out of the five studies mentioned above [21,27,28,29].

Olivieri et al. created 3D printed models from 10 patients who underwent congenital cardiac surgery due to various cardiac and vascular anomalies [26]. They presented the 3D models to 70 clinicians including 22 physicians, 38 critical care nurses, and 10 ancillary providers. At completion of the cardiac surgeries, all participants underwent a training session of simulating intra- and post-operative care using 3D printed heart models. The use of 3D printed models was found to be more effective than standard verbal hand off with average score of 8.4 out of 10. In total, 90% of participants scored it very highly with regard to the efficacy of 3D printed models in improving cardiac anatomy understanding, surgical understanding, and ability to manage CHD clinically.

Two other studies reported utilizing 3D printed heart models in the diagnostic management of patients with CHD [21,27]. Hoashi et al. created 20 3D printed heart models for the purpose of preoperative simulations of cardiac surgeries [27]. Despite realistic and expensive models being produced (each model costs between $2000 and $3000), this study mainly focused on findings related to patient’s cardiac surgery outcomes, while the value of 3D printed models was briefly mentioned in some sample cases. Specifically, authors concluded that 3D printed heart models did not reduce cardiopulmonary bypass time. In contrast, Valverde et al. conducted a multi-center study and performed both quantitative and qualitative assessments of the role of 3D printed models in clinical decision-making in patients with complex CHD [21]. Forty patients recruited from 10 international centers were included in this prospective study with 3D models fabricated using CT or MRI images. 3D printed models were assessed as to whether they changed the surgical decision (from conservative management to surgical intervention) and whether the surgical plan was modified. In more than half of the cases (52.5%), 3D printed models did not result in any change to the surgical decision. However, 3D printed models showed significant clinical impact on redefining the surgical approach in 47.5% cases. In 25% of cases, after inspection of 3D printed models, the surgical plan was modified with conservative management changed to surgery. As the only multi-center study available in the literature, this study shows the impact of 3D printed models on deciding the best surgical approach. However, more similar studies are desirable to validate this.

The other two studies are based on single-center experience reporting the clinical impact of 3D printed models in CHD treatment outcomes [28,29]. Zhao et al. divided 25 patients with complex DORV into two groups, 8 in the 3D printing group and 17 in the control group, with all patients undergoing cardiac surgery [28]. The intensive care unit stay time and mechanical ventilation time in the 3D printing group was significantly shorter than in the control group (*p* < 0.05). Although the operative duration, cardiopulmonary bypass time, and aortic cross-clamping time in the 3D printing group was shorter than the control group, this did not reach statistical significance (*p* > 0.05). Similar findings are reported by Ryan and colleagues [29]. The authors presented their single-site three-year experience of using 3D printed models for managing CHD cases. Of 164 models fabricated for different purposes, 79 models covering a range of CHD complexities were selected for surgical planning. When compared to the standard care (without anatomical models) group, the 3D printed heart model group was found to have shorter mean duration in the operative room and lower 30-day readmission and mortality rates. However, it is worthwhile to note that it did not reach statistical significance, and it is likely due to limited study sizes for each CHD types. These reductions in durations could contribute to lower morbidity and mortality associated with management of CHD, although this needs to be validated by further studies. One example would be by investigating the impact of 3D printed models on 30-day post-operative outcome.

## 6. 3D Printed Models in Doctor–Patient Communication

Physician–patient relationship and working alliance plays a crucial role in improving patient adherence, level of satisfaction, and treatment outcomes [34]. Due to complexity and variations of cardiac anatomy in CHD, it is especially challenging in achieving good physician–patient communication (physicians specifically refer to cardiologists and cardiac surgeons in the situation of managing patients with CHD) [35]. Traditional approaches of using diagrams or image visualizations for explanation of complicated cardiac pathologies do not allow doctors to effectively communicate to patients or parents because of difficulty in interpreting 3D conceptualization of spatial relationship between cardiac structures. 3D printed models are able to eliminate this limitation as observers have no restriction in appreciating the spatial relationship between cardiac structures in all dimensions, thus improving doctor–patient communication.

A study by Biglino et al. first attempted to quantify the benefit of 3D printed models in doctor–patient communication [36]. Ninety-two parents of patients with CHD were randomly allocated to two groups with 45 assigned to the model group using 3D printed heart models during their visit, and 52 to the control group with no models during consultation. Parents were asked to complete two questionnaires: A first brief questionnaire before their child’s consultation and a second brief questionnaire after the consultation with regard to understanding of their child’s heart condition, identification of cardiac defects, and clarity of planned intervention or procedure. Both cardiologists and parents rated the 3D printed models as very useful. Despite the improvement in doctor–parent communication, 3D printed models did not lead to improving parents’ knowledge and understanding of their child’s heart condition. Furthermore, consultations using the 3D printed models were found to be longer than those without the models (21 ± 10 vs. 16 ± 7 min, *p* = 0.02), although this did not show significant impact on overall duration of the visits.

**Figure 6:**
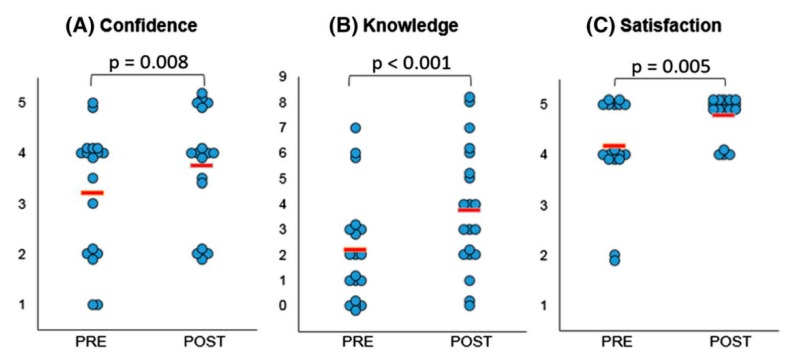
Statistically significant differences were noted in confidence (**A**), knowledge (**B**), and satisfaction (**C**) amongst participants comparing responses before (“Pre”) and after (“Post”) their medical consultation. (**A**) 1 refers to not at all confident and 5 very confident. (**B**) Each point represents a point in knowledge, as marked based on the correct name of primary diagnosis, correctly identified keywords, and correct use of diagrams. (**C**) 1 indicates very dissatisfied and 5 very satisfied. The red lines indicate average score. Reprinted with permission under the open access from Biglino et al. [36].

**Figure 7:**
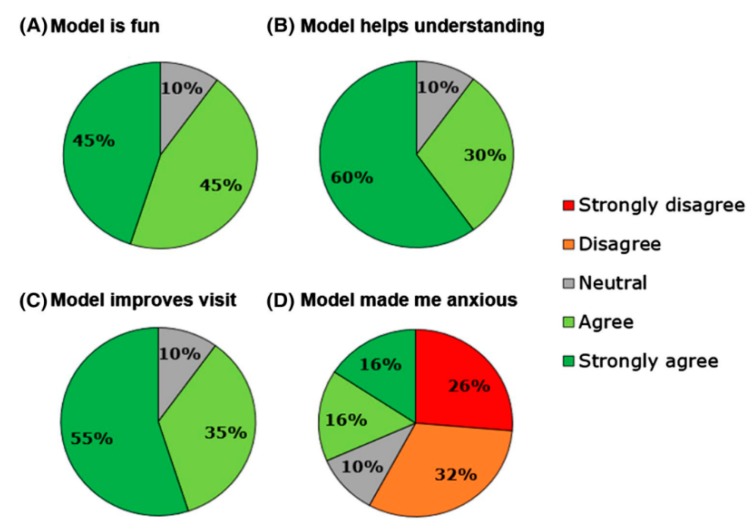
Participants’ response to different statements on the usefulness of 3D printed models. Reprinted with permission under the open access from Biglino et al. [36].

The same group conducted another study determining the impact of using 3D printed heart models on facilitating consultations between doctor and young people with CHD [37]. Twenty adolescent patients with CHD (age range 15–18 years) were included in this study with use of the same approach as stated in the previous study involving completion of two questionnaires, pre and post-consultations with their doctors. Positive responses were found in the study with use of 3D printed models with significant improvements in their knowledge of CHD (*p* < 0.05), confidence in explaining conditions to others (*p* < 0.001), and overall satisfaction (*p* < 0.05) (Figure 6). The majority of participants indicated that the 3D printed models improved their clinical visits, however, 30% of them expressed their concern of feeling more anxious about their heart condition with use of the 3D models (Figure 7).

More research is needed to investigate the clinical translation of 3D printed heart models for doctor–patient/parent communication with involvement of different stakeholders including patients, parents, families, clinicians, and other healthcare professionals such as nurses and ancillary providers [28]. Further studies are also required to address the limitations of lack of evidence of clinical follow-up with regard to the impact of 3D printed models on patient’s lifestyle and eventually patient outcomes.

## 7. Summary and Future Research Directions

Personalized 3D printed models of CHD are changing the current practice in the diagnostic management of patients with CHD. 3D printed models have demonstrated advantages over traditional image visualizations in the assessment of complex cardiac structures as observers are able to appreciate various CHD conditions with more confidence. Three main applications of 3D printed heart models have been discussed in this review, including medical education and training, pre-surgical planning and simulation, and doctor–patient communication. Despite attractiveness of 3D printed realistic models and promising results associated with their applications, more scientific evidence with high statistical power is needed before 3D printing is widely used in clinical practice. In addition to the lack of large-scaled studies (prospective and multi-center studies), some limitations should be addressed with future technical developments so that 3D printing will be more practicable in medical applications.

One of the main limitations in generating 3D printed heart models lies in image post-processing and segmentation of cardiac imaging data, which is exceptionally time-consuming and requires expertise in image analysis. The duration needed to complete the segmentation and image post-processing is highly dependent on segmentation software tools (whether it is powerful enough for automatic segmentation), and researcher’s familiarity with the software, as highlighted by two systematic reviews and other review articles (Table 2) [29,35,38,39,40]. This operator-dependent process is inevitably associated with interobserver and intraobserver variability. This limitation could be resolved with the use of artificial intelligence or machine learning algorithms which are increasingly used in the domain of cardiovascular disease by providing automated image segmentation of coronary plaque lesions or coronary lumen [41,42,43,44], thus improving the workflow efficiency from image acquisition to 3D printing.

**Figure 8:**
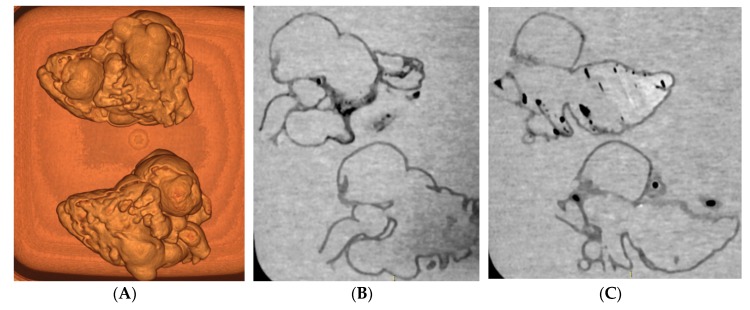
CT scan of 3D printed heart models created using different printing materials. (**A**) 3D volume rendering showing the 3D printed models without contrast medium (top: Tango Plus material, bottom: TPU material). (**B**,**C**) Coronal multiplanar reformatted contrast-enhanced CT images showing 3D printed models with Tango Plus (left) and TPU (right) materials. Air bubbles are noticed in the model with TPU material. TPU—thermoplastic polyurethane. Reprinted with permission under the open access from Lau et al. [45].

**Figure 9:**
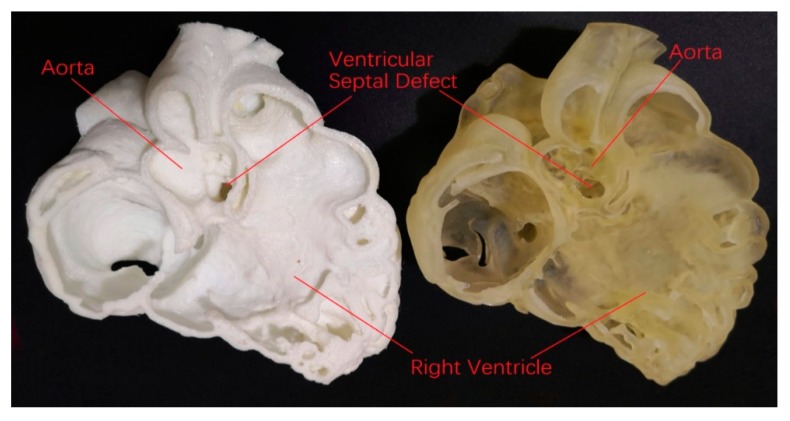
Comparison of low-cost (left image) with high-cost (right image) 3D printed heart model with similar accuracy in delineating cardiac anatomy and ventricular septal defect.

High 3D-printing costs represent another obstacle, however this is being resolved with the use of low-cost 3D printing materials, provided that the accuracy of the 3D printed models is not affected. A recent study has demonstrated the feasibility of creating accurate 3D printed models using low-cost as opposed to high-cost material ($50 vs. $300) for delineation of cardiac anatomy and defects (Figure 8 and Figure 9) [45]. With further cost reductions in 3D printers and printing materials in the near future, personalized 3D printed heart models will become more affordable to patients with CHD. Table 3 shows different types of 3D printers and printing materials that are available in the 3D printing of cardiac models and strengths and weaknesses of these models corresponding to each type of 3D printers [36,46,47,48,49].

Very few materials that are currently used for creating 3D printed heart models represent elastic properties similar to human tissue which allow for performance of realistic surgical simulation such as cutting and suturing of cardiac structures. Despite softness of the Tango Plus material as shown in Figure 9, the mechanical properties of these materials are still different from biologic heart tissues. Furthermore, current 3D printers generate a static heart model instead of a dynamic organ, therefore, allowing for assessment of morphological cardiac features rather than hemodynamics of the cardiovascular system. Future developments in printing technologies should aim to produce 3D printed dynamic heart models which enable detection of both anatomic and physiological changes during the cardiac cycle [11,39,50]. 3D bioprinting represents another major advancement with the capability of printing biomaterials, 3D printed tissue scaffolds, and 3D printed functional vascular networks [51,52,53]. Bioprinting of patient-specific heart tissues will broaden applications of 3D printing in CHD, although many challenges need to be overcome before it can be translated to clinical applications [54,55].

**Figure 10:**
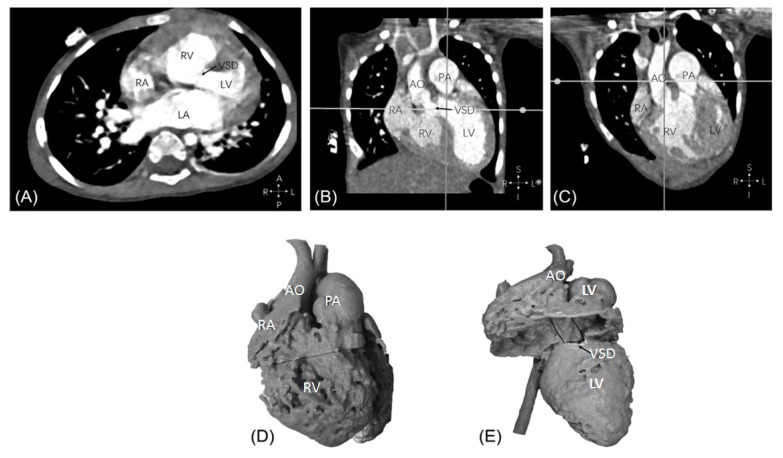
Example of double outlet right ventricle with aorta and pulmonary artery arising from the right ventricle and perimembranous ventricular septal defect from computed tomography images (**A**–**C**). Anterior view of the 3D printed heart model, aorta, and pulmonary artery are side-by-side with both arising from the right ventricle (**D**). Perimembranous VSD remoted from the arteries. Position of potential intracardiac tunnel from the left ventricle to the aorta is shown as the solid lines (**E**). AO—ascending aorta; LA—left atrium; LV—left ventricle; PA—pulmonary artery; RA—right atrium; RV—right ventricle; VSD—ventricular septal defect. Reprinted with permission from Zhao et al. [32].

**Figure 11:**
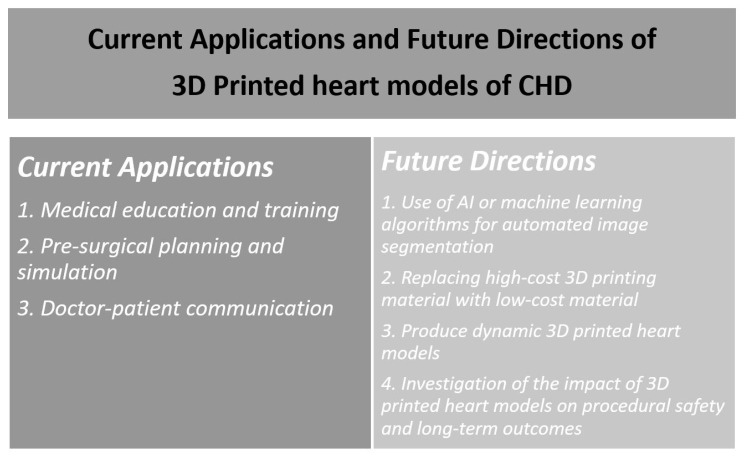
Summary of current applications and future research directions of 3D printing in congenital heart disease. 3D—three-dimensional; CHD—congenital heart disease; AI—artificial intelligence.

Currently, no guidelines or recommendations are available regarding the standardized use of 3D printed models in CHD patients. Use of 3D printed models is limited to complex CHD such as DORV (Figure 10) and ToF as evidenced by anecdotal reports and case series [16,21,22,23,31,32,33,56,57]. Other clinical benefits of using 3D printed heart models such as its impact on procedural safety and long-term outcomes are still yet to be investigated. Future research should focus on these areas as they will contribute to the development of clinical recommendations of using 3D printed models routinely in medical practice, therefore having great impact on the treatment of congenital heart disease. Figure 11 presents a summary of the current applications and future directions of 3D printing in CHD.

## Figures and Tables

**Table 1:** Study characteristics of randomized controlled trials and multi- and single-center studies.

Authors	Study Design	Sample Size and Participants	Types of CHD	Key Findings
Loke et al. 2017 [22]	RCT: study group was presented with 3D printed models, while control group with 2D images	35 pediatric residents:18 in study group and 17 in control group	Tetralogy of Fallot (ToF)	3D printed models resulted in significantly higher satisfaction scores than 2D images (*p* = 0.03). 3D printed models improved residents’ self-efficacy scores in managing ToF, although this did not reach significant difference when compared to 2D images (*p* = 0.39).
Su et al., 2018 [23]	RCT: study group participated in teaching seminar including 3D printed models, while control group only attended teaching seminar without having 3D models	63 medical students: 32 in study group and 31 in control group	Ventricular septal defect (VSD)	Significant improvement in VSD learning and structure conceptualization in the study group compared to the control group (*p* < 0.05).
White et al., 2018 [24]	RCT: study group was given 3D printed models in addition to lectures, while control group received only the lectures	60 pediatric residents:31 in study group and 29 in control group	VSD and ToF	3D printed models of CHD improved residents’ knowledge and confidence in managing complex CHD such as ToF but did not seem to improve simple CHD such as VSD.
Olivieri et al., 2016 [26]	Single-center report of 3D printed models for training and simulation	10 3D printed models, 70 clinicians participated in the training sessions	Cardiac and vascular anomalies	3D printed models can be used as a simulation training tool for multidisciplinary intensive care providers by enhancing their anatomic knowledge and clinical management of CHD patients.
Hoashi et al., 2018 [27]	Single-center experience	20 cases	DORV and other cardiac anomalies	3D printed heart models improved understanding of the relationship between intraventricular communications and great vessels. Further, 3D printed models allowed simulation of cardiac surgeries by creating intracardiac pathways, thus providing benefits to inexperienced cardiac surgeons.
Valverde et al., 2017 [21]	Multi-center study consisting of 10 international centers	40 patients with complex CHD	DORV (50%) and other cardiac anomalies	3D models were accurate in replicating anatomy. 3D models refined the surgical approach in nearly 50% cases. 3D models resulted in significant change in the surgical plan in 24% of cases.
Zhao et al., 2018 [28]	Single-center experience	25 patients with 8 in 3D printing group and 17 in control group	DORV	3D printed models showed high accuracy in measurements of aortic diameters and the size of VSD when compared to original CT data. 3D printed models significantly reduced ICU time and mechanical ventilation time (*p* < 0.05).
Ryan et al., 2018 [29]	Single-center experience	Of 928 cardiothoracic surgeries, 164 3D models were printed for various purposes	DORV, ToF and other cardiac anomalies	3D printed models reduced mean time in the operating room and 30-day readmission and mortality rates when compared to the standard of care.

CHD—congenital heart disease, DORV—double outlet right ventricle, ICU—intensive care unit, RCT—randomized controlled trial.

**Table 2:** Summary of systematic reviews of 3D printed models in congenital heart disease.

Authors	Number of Studies Analyzed	Review Purpose	Key Findings
Batteux et al., 2019 [38]	NR	Accuracy and reliability of 3D printed models in surgical planning in complex CHD	3D printed models improve understanding of complex cardiac anatomy and disease and can be used to guide surgical planning.
Lau and Sun 2018 [29]	28	Clinical value of 3D printed models in CHD	3D printed models accurately replicate cardiac anatomy and pathology and are shown to be valuable in preoperative planning and simulation of cardiac procedures.

NR—Not reported.

**Table 3:** Summary of different types of 3D printing technologies and corresponding 3D printed heart models. Adapted from References [36,46,47,48,49].

3D Printing Technologies	Printing Materials	Advantages	Disadvantages	3D Printed Heart Models	
				Strengths	Weaknesses
Stereolithography (SLA)	Photopolymers	Large part size	High cost, moderate strength	High detail and accuracy, smooth surfaces	Low tensile strength
Polyjet (PJ)	Photopolymers	Variety of materials including multi-colored materials	Slow speed, high cost	High accuracy with flexibility, durability, and translucency	Low tensile strength
Selective Laser Sintering (SLS)	Powder materials	Large part size, variety of materials and good strength	High cost, low resolution	Moderate accuracy	Inferior anatomical details
Binder Jetting (BJ)	Powder materials	Very low cost, variety of materials, relatively fast, does not use heat	Slow speed, fragile parts with limited mechanical properties	NR	Low accuracy
Fused Deposition Modeling (FDM)	Thermoplastic materials	Low cost, variety of materials, good strength	Slow speed and a scaffold is needed to support the object during printing	Moderate accuracy, more suitable for medical devices	Limited values in surgical and anatomical models
